# A Comprehensive Report of Intrinsically Disordered Regions in Inherited Retinal Diseases

**DOI:** 10.3390/genes14081601

**Published:** 2023-08-08

**Authors:** Karen E. Lee, Jose S. Pulido, Mariana M. da Palma, Rebecca Procopio, Robert B. Hufnagel, Margaret Reynolds

**Affiliations:** 1 Department of Pediatric Ophthalmology and Strabismus, Wills Eye Hospital, Philadelphia, PA 19107, USA; klee@willseye.org (K.E.L.); rprocopio@willseye.org (R.P.); 2 Retina Service, Wills Eye Hospital, Philadelphia, PA 19107, USA; jpulido@willseye.org; 3 Department of Ophthalmology, Federal University of São Paulo, UNIFESP, São Paulo 04023-062, SP, Brazil; marimatioli@gmail.com; 4 Medical Genetics and Ophthalmic Genetics Unit, National Eye Institute, Bethesda, MD 20892, USA; robert.hufnagel@nih.gov; 5 Department of Ophthalmology and Visual Sciences, Washington University School of Medicine, St. Louis, MO 63110, USA

**Keywords:** genetics, IDR, IRD, proteins, intrinsically disordered regions, inherited eye diseases, RetNet

## Abstract

Background/purpose: A comprehensive review of the degree of disorder in all genes in the Retinal Information Network (RetNet) Database is implicated in inherited retinal diseases (IRDs). Their association with a missense variation was evaluated. Methods: IRD genes from RetNet were included in this study. Publicly available data on the genome aggregation database (gnomAD) were used to analyze the number of total and pathogenic missense variants. Metapredict, an accurate and high-performance predictor that reproduces consensus disorder scores, was used to calculate disorder. Main outcome measures: The main outcome measures were percent disorder, percent pathogenicity, number of total missense variants, and percent total missense variation. Results: We included 287 RetNet genes with relevant data available from gnomAD. Mean percent disorder was 26.3% ± 26.0%, mean percent pathogenicity was 5.2% ± 11.0%, mean number of total missense variants was 424.4 ± 450.0, and mean percent total missense was 50.0% ± 13.4%. The percent disorder followed a bimodal distribution with the highest number of occurrences in the 0 to 10th disorder decile. The five outlier proteins in the first disorder decile with a higher-than-expected number of total missense variation were identified (HMCN1, ADGRV, USH2A, DYNC2H1, LAMA1, and SLC38A8). When excluded, % total missense was significantly associated with percent disorder (R = 0.238 and *p* = 0.0240). Conclusions: This novel study examining all genes implicated in IRDs found that the majority genes had a disorder in the 0 to 10th decile and were relatively intolerant to missense variation. This may have future utility when interpreting variants of undetermined significance and missense variants.

## 1. Introduction

Inherited retinal diseases (IRDs) are a clinically and genetically heterogenous group of visually debilitating diseases caused by pathogenic variation in proteins critical for retinal function [1]. Over 300 causative genes have been implicated in IRDs, and these genes encode a spectrum of proteins including structural and transmembrane proteins, phototransduction proteins, and other regulatory proteins involved in the visual cycle [2]. Intrinsically disordered regions (IDRs) are made of protein sequences that lack hydrophobic amino acids and do not form a hydrophobic core or 3D structure. They lack stable structures in isolation and instead exist as an ensemble of conformations that varies depending on other protein–protein interactions. IDRs play crucial roles in many biological processes, including cell signaling and regulation [3]. Therefore, it is not surprising that they have been linked to the pathogenesis of neurodegenerative diseases.

Recently, Lee et al. showed that the IRD proteins that exhibit a high degree of disorder tolerate a higher degree of missense variation and that these proteins also exhibit a lower amount of pathogenic missense variants with respect to total missense variants [4]. These findings were observed in 14 genes implicated in inherited retinal diseases (IRDs), which were categorized into four groups: (1) proteins with overrepresentation of missense variants (SAMD11, ALMS1, WFS1, RP1L1, KCNV2, and ADAMTS18), (2) transmembrane transport (CNGB1, CNGA1, TRPM1, ABCA4, BEST1, and KCNV2), (3) internal or structural proteins of the photoreceptors that are essential in visual function (RHO and RPE65), and (4) secreted proteins (TIMP3 and ADAMTS18) [4]. IDRs are known to affect channel protein function [5].

Though these initial investigations yielded interesting results, we wanted to further evaluate these findings in a larger number of proteins. We therefore examined all proteins in the RetNet Database, which provides a list of 324 genes and loci implicated in IRDs. We investigated the degree of disorder in each protein and compared it to the number of nonsynonymous missense variants. Through this analysis, we identify outlier proteins that do not exhibit predicted relationships between disorder and missense variation. Further investigation of these genes may be warranted to examine how their defining characteristics may be associated with protein function and disease.

## 2. Materials and Methods

### 2.1. Gene Identification

Genes listed in the Retinal Information Network online resource (https://sph.uth.edu/retnet/ accessed on 16 October 2022) were included in this study. The mitochondrial genome was excluded from this analysis.

#### Databases and Metrics

The amino acid sequences of each protein used in this study were obtained through the National Institutes of Health (NIH) National Library of Medicine Protein search tool. The Mane Select transcript was selected for each protein. The sequences were then run through Metapredict (version 2.3) to plot the intrinsically disordered regions (IDRs) as previously described [4]. Because multiple predictors are available, consensus scores have emerged to interpret the outcomes of multiple independent disorder predictors. Metapredict uses deep-learning-based predictors of consensus scores from 12 proteomes to determine IDRs [4]. We calculated the percent disorder defined as the percentage of the number of amino acids in IDRs as a fraction of the total number of amino acids in the protein. 

The resource gnomAD was used to examine the number of pathogenic missense variants and total missense variants for each protein as previously described [4], and gnomAD is currently the largest and most widely accessed reference population database. It provides rapid variant analysis. ClinVar is maintained by the National Institutes of Health and is a public archive of human genetic variants. ClinVar variants noted in the November 2022 version included in gnomAD were used to examine the number of pathogenic missense mutations as previously described. 

We calculated percent pathogenicity defined as the percentage of pathogenic missense variants as a fraction of the number of total missense variants. We calculated the percent total missense defined as the percentage of the total number of missense variants as a fraction of the total number of amino acids in the protein. 

Regression analysis was conducted using SPSS Statistics with significance set at *p* < 0.05.

## 3. Results

Of the 336 genes and loci included in the RetNet Database, 7 mitochondrial genes were excluded as well as 42 additional entries due to the (1) lack of relevant data available on gnomAD or the (2) historical linkage regions synonymous with genes that have since been identified, leaving 287 available for inclusion (Supplementary Table S1). 

The mean percent disorder in all proteins was 26.3 ± 26.0 (range 0–100), mean percent pathogenicity was 5.2 ± 11.0 (range 0–87), mean number of total missense variants was 424.4 ± 450.0 (range 10–3130), and mean percent total missense was 50.0 ± 13.4 (range 3.1–100). 

**Figure 1:**
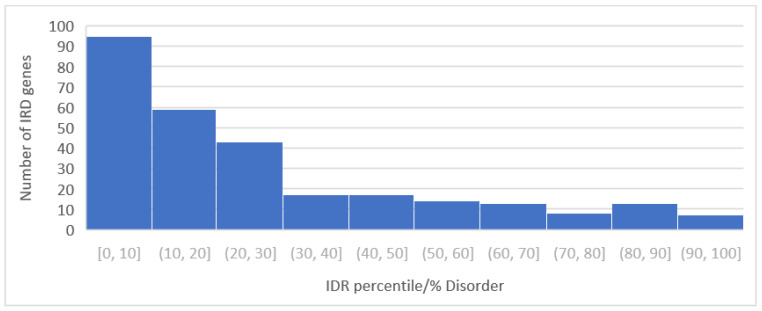
Distribution of percentile of intrinsically disordered regions (IDRs) in all proteins showing highest distribution in 0,10 decile and lowest in 90,100 decile.

**Figure 2:**
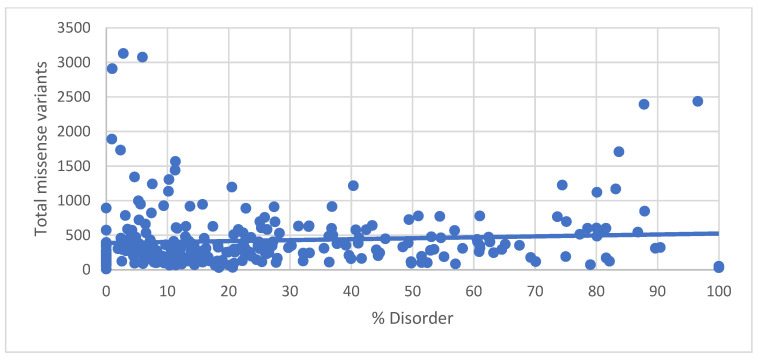
% disorder versus total number of missense variants. % disorder = (number of amino acids in IDRs/total number of amino acids in protein) × 100. Total number of missense variants is obtained from gnomAD browser.

The percentile distribution of IDRs in all proteins is shown in Figure 1, which plots the number of proteins with respect to the percent disorder organized by decile. The highest number of occurrences was in the 0 to 10th disorder decile, and the lowest distribution was in the 90th to 100th disorder decile. We previously showed that proteins with a high degree of disorder tolerated the greatest amount of missense variation [4], so we plotted the percent disorder of all proteins in the RetNet Database with respect to the total number of missense variants (Figure 2). Regression analysis between disorder and total missense variants showed R = 0.079 and *p* = 0.18, between percent disorder and percent pathogenicity showing R = 0.024 and *p* = 0.68, as well as between % total missense and % disorder showing R = 0.062 and *p* = 0.301. 

**Figure 3:**
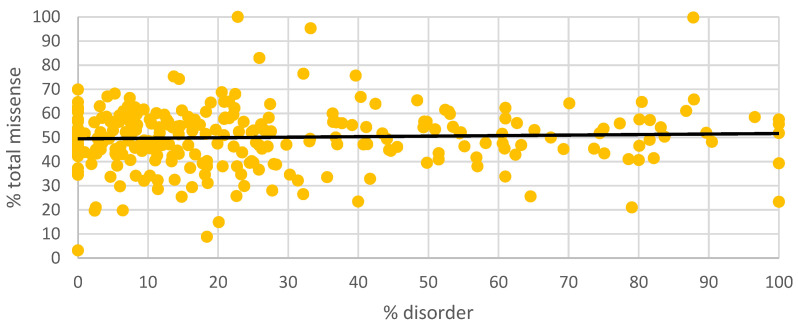
% disorder versus % total missense. % disorder = (number of amino acids in IDRs/total number of amino acids in protein) × 100. % missense = (number of total missense variants/total number of amino acids in protein) × 100. Total number of missense variants is obtained from gnomAD browser.

**Figure 4:**
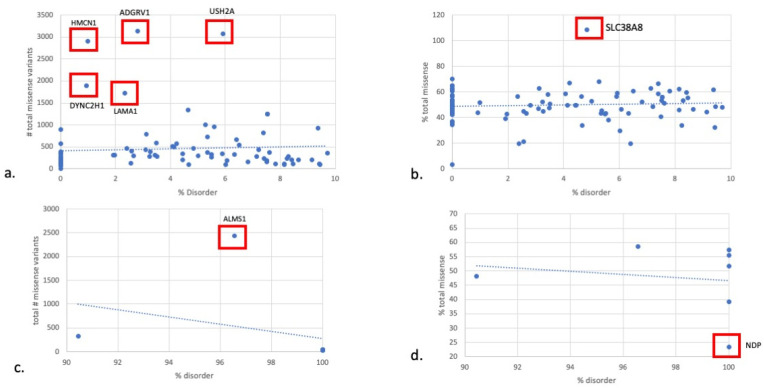
% disorder versus # of total missense variants in 0–10% disorder decile. (**a**) % disorder versus # total missense variants in 0–10% disorder decile. (**b**) % disorder versus % total missense in 0–10% disorder decile. (**c**) % disorder versus # of total missense variants in 90–100% disorder decile. (**d**) % disorder versus % total missense in 90–100% disorder decile. % missense = (number of total missense variants/total number of amino acids in protein) × 100. % disorder versus total number of missense variants. % disorder = (number of amino acids in IDRs/total number of amino acids in protein) × 100.

Figure 3 displays a scatterplot of percent disorder with respect to percent total missense. To interrogate the differences between proteins at the 0 to 10th disorder decile and 90th to 100th disorder decile, we plotted the percent disorder with respect to the total number of missense variants and the percent total missense for each group (Figure 4a–d). Proteins existing as outliers in the 0–10th decile of the disorder group include HMCN1, ADGRV, USH2A, DYNC2H1, LAMA1, and SLC38A8. Removing the five outliers from the 0–10th disorder decile revealed mean total missense variants of 336.5. 

**Figure 5:**
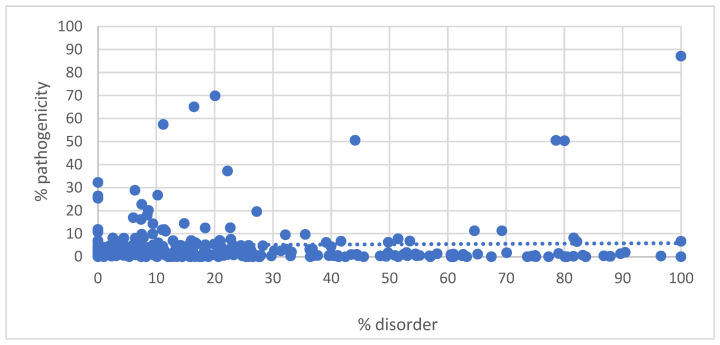
% disorder versus % pathogenicity. % disorder = (number of amino acids in IDRs/total number of amino acids in protein) × 100. % pathogenicity = (number of pathogenic missense variants/total number of missense variants) × 100.

**Figure 6:**
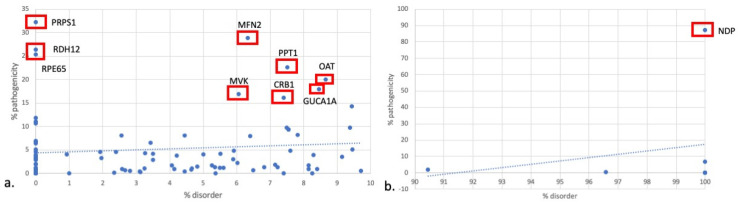
% disorder versus % pathogenicity in (**a**) 0–10% disorder decile and (**b**) 90–100% disorder decile. % disorder = (number of amino acids in IDRs/total number of amino acids in protein) × 100. % pathogenicity = (number of pathogenic missense variants/total number of missense variants) × 100.

Percent disorder with respect to percent pathogenicity for all proteins is shown in Figure 5. Proteins in the 0 to 10th disorder decile exhibited a mean percent pathogenicity of 5.2. When the outliers were excluded, proteins in the 0 to 10th disorder decile exhibited a mean percent pathogenicity of 8.6. Proteins in the 90th to 100th disorder decile exhibited a mean percent pathogenicity of 13.7. On the exclusion of NDP, the percent pathogenicity for proteins in the 90th to 100th disorder decile was reduced to 10%. Outlier proteins in the 0 to 10th disorder decile include PRPS1, RPE65, RDH12, MFN2, MVK, PPT1, OAT, CRB1, and GUCA1A. These proteins all exhibited pathogenicity above 15% (Figure 6). 

Regression analysis in the 0 to 10th disorder decile, for all proteins, between total missense variants and percent disorder showed R = 0.059 and *p* = 0.6, between percent pathogenicity and percent disorder showing R = 0.007 and *p* = 0.3, as well as between percent total missense and percent disorder showing R = 0.069 and *p* = 0.508. These values did not change significantly with the outliers removed. In the 90th to 100th disorder decile, the regression between total missense variants and percent disorder showed R = 0.306 and *p* = 0.504 between percent pathogenicity and percent disorder showing R = 0.225 and *p* = 0.627, as well as between percent total missense and percent disorder showing R = 0.151 and *p* = 0.747. 

When removing the five protein outliers in the 0 to 10th disorder decile, the regression between % total missense and percent disorder showed R = 0.238 and *p* = 0.024.

## 4. Discussion

We show that in the cohort of RetNet encoded IRD proteins, the average percent disorder was approximately 26%, with most proteins concentrated in the 0 to 10th decile of disorder. Our previously published work showed that internal or structural proteins of photoreceptors were the most ordered and have low pathogenic missense to total missense variants ratio. In the 0 to 10th disorder decile where the majority of our proteins were concentrated, HMCN1, DYNC2H1, ADGRV1, LAMA1, and USH2A exhibited low disorder but had an unexpectedly high number of total missense variants. We deemed these five proteins “outlier proteins”, four of which are internal or structural proteins. HMCN1 is an extracellular matrix protein sharing similarities to fibulins and has been linked to retinitis pigmentosa. DYNC2H1 is a dynein protein that is involved in retrograde transport in cilia, and its variants have been known to cause Leber congenital amaurosis [6]. USH2A and LAMA1 are both extracellular matrix proteins. When the outlier proteins were removed from the main analysis, the regression between percent disorder and total missense variation showed a stronger positive correlation and became significant (*p* = 0.024). Consistent with previously published reports [7] and our previous work, this suggests that regions with more disorder do tolerate a greater degree of missense variation. 

With respect to SLC38A8, the one protein in the 0–10% decile disorder which was an outlier for % total missense, *SLC38A8* codes for a proton antiporter. It has been implicated in foveal hypoplasia. It had a higher number of missense variants relative to the number of amino acids compared to what would be expected for a percent disorder. However, its overall percent pathogenicity is low at 1.48%, which may mean that the functional effect of many of these missense variants is benign [8]. 

In regards to the five proteins in the 0 to 10th disorder decile with an outlier number of total missense variants, USH2A mutations are the most frequent cause of inherited retinal dystrophy, including Usher Syndrome type II and non-syndromic retinitis pigmentosa [9]. *USH2A* encodes usherin, a transmembrane protein of 5202 amino acids that contains laminin EGF motifs, a transmembrane domain, and fibronectin repeats [10]. It is predominantly expressed in photoreceptors. One would expect a low toleration of missense variation with only 5.9% disorder, but it has the second highest number of total missense variation (3076). It is possible that the functional effect of a large portion of the missense variants is benign as the percent pathogenicity was relatively low at 4.8%. *ADGRV1* is also a cause of Usher Syndrome type II and share many characteristics of functional and structural impairment with *USH2A* [9]. Both *USH2A* and *ADGRV1* belong to the same protein complex, are located at the periciliary region between the inner and outer segment of photoreceptors, and are thought to be important for stabilizing the connecting cilium [11]. ADGRV1 and USH2A retinopathy produce indistinguishable clinical profiles, suggesting that their loss of function produces similar effects in the retina [10]. 

Lastly, there are 10 proteins with a greater amount of pathogenicity expected relative to the percent disorder. First, NDP has a high degree of disorder (100%) despite having a relatively high percent pathogenicity (87.1%). The reasons for this are unclear, though it is interesting to note that only exons 2 and 3 are translated [12]. PRPS1, RPE65, and RDH12 have 0 amino acids in IDRs, so using IDRs to inform diagnostics surrounding mutations affecting these proteins may be less helpful. However, it makes sense that these proteins do not tolerate mutations well given that their percent disorder is minimal. Therefore, they do follow our hypothesis. 

There are several limitations to this study. Metapredict utilizes a machine learning approach known as knowledge distillation, whereby a computationally cheap model is trained with data generated with computationally expensive models, which can have limitations [13]. For instance, our analysis demonstrated that genes in the first quartile in size exhibited significantly higher disorders (24.3% versus 6.3% and *p* = 0.002). Smaller proteins may be subject to more difficulty with prediction, and thus, analyzing the relationship between protein size and disorder prediction may be an area of future investigation.

## 5. Conclusions

Of the 287 genes studied from the RetNet Database, we found that most IRD proteins were concentrated in the 0 to 10th disorder decile and thus relatively intolerant of missense mutation. Removing outliers in the 0 to 10th disorder decile showed that proteins with a higher percent disorder exhibited significantly more total missense variation (*p* = 0.024). We found lower percent pathogenicity in the 0 to 10th disorder decile. This study is the first to our knowledge to examine the disorder and gene variant profiles of all RetNet genes available. IDR should be considered when interpreting variants of undetermined significance and missense variants.

## Data Availability

Data available upon request to corresponding author.

## Supplementary Materials

The following supporting information can be downloaded at: https://www.mdpi.com/article/10.3390/genes14081601/s1, Table S1: Protein Name.
