# Supplementary material for: A Comprehensive Report of Intrinsically Disordered Regions in Inherited Retinal Diseases
Source: Genes (Basel). 2023 Aug 8;14(8):1601. doi: 10.3390/genes14081601 (PMC10454668; doi:10.3390/genes14081601)
Supplement: Supplementary file 1 [file genes-14-01601-s001.zip › genes-2495466-supplementary.pdf]

Supplementary Table S1: Protein Name

DHDDS
RPE65
GNAT2
DRAM2
OR2W3
IFT172
BBS5
GNAT1
ARL6
CLRN1
WDR19
HARS
GUCA1B
PEX7
PEX2
HK1, RP79;
RGR, RP44;
ARL3;
CAPN5, ADNIV, HTRA3, VRNI;
TMEM126A, OPA7;
GNB3, CSNB1H;
RDH5, RDH1;
CCT2, CCTB;
RCBTB1, RCBT1;
IFT27, BBS19;
RDH11
RDH12
CIB2
RLBP1
BBS2
FSCN2
PGK1
PRPS1
DYNC2H1, SRTD3;
HMCN1
ACO2, ACONM, ICRD, OPA9;
BBS7
LAMA1
PRPF8

KLHL7
MTTP
IFT81, CDV1;
ADGRV1
TLR3
RBP3, IRBP, RP66;
ARSG
PDE6C, ACHM5, COD4, PDEA2;
CYP4V2
CFH
MKKS
PDE6A
EMC1
PDE6B
PROS1
SAG
NBAS
ELOVL1
SLC38A8
MKS1
IFT140
C3
TLR4, ARMD10;
CFB
IMPDH1
CEP290, BBS14, JBTS5, LCA10, NPHP6, MKS4,
SLSN6;
BBS1;
USH2A
LZTFL1
MVK;
MFN2
CDH23, DFNB12, USH1D;
GRM6
EXOSC2;
TRNT1
BBS9/PTHB1
CRB1
ROM1;
HTRA1, ARMD7, PRSS11;

PPT1
MYO7A, DFNB2, USH1B;
USH1E; myo7a
BBS10, FLJ23560;
OPA3
RAB28
REEP6
TUBGCP4
MFSD8
OPN1SW
GUCA1A
OAT;
FBLN5
ABCC6
RP2
RBP4;
C2
RTN4IP1
CEP250
ABCA4
KCNJ13
TMEM216, CORS2, JBTS2, MKS2;
PHYH, PAHX, RDPA;
GRK1, RHOK, RK;
OPA1
TTPA
RHO
EYS
BBS12
DMD
TIMP3, SFD;
SNRNP200
GUCY2D
IDH3B
IQCB1
PLA2G5
ASRGL1;
POMGNT1
RS1
PROM1

NPHP3
ELOVL4
STGD2 renamed to ELOVL4
NEK2
SEMA4A
ADAMTS18
CTNNA1
HGSNAT
NPHP1
TRIM32, BBS11, HT2A;
MAPKAPK3
TIMM8A
TTC8
OPA5;
AIPL1
LRP5, EVR4, HBM, OPPG;
RGS9BP
CODA1; MMP19
JAG1
KIF11, EG5, HK5P, KNSL1, MCLMR, TRIP5;
ABHD12
PRPH2
CA4
TSPAN12
LRAT
CACNA2D4, RCD4;
ARL2BP
OPN1LW
SDCCAG8
DYNC2I2, SRTD11, WDR34;
OPN1MW
RNANC; ATOH7
ITM2B, ABRI;
WFS2/ CISD2
SPP2
POC1B, CORD20;
FZD4, EVR1, FEVR;
NR2F1
PCDH15, DFNB23, USH1F;
BBIP1, BBIP10, BBS18;

MERTK
CEP19
PANK2
CLN3
USH1C, DFNB18;
BBS4
CERKL
NMNAT1
CDHR1, CORD15, PCDH21, RP65;
MFRP, NNO2, MCOP2;
PRPF6
AFG3L2
WFS1
SLC25A46
KIF3B
FLVCR1
CDH3
CHM
PCYT1A
CNGA1
ADAM9
SLC7A14
CNNM4
RPGRIP1L
KCNV2
UNC119
DTHD1
CC2D2A
GNPTAG
CLUAP1
PEX1
MIEF1;
WDPCP
CNGA3
NPHP4
GPR125/ADGRA3
ADIPOR1
TEAD1, AA, TCF13, TEF1;
PNPLA6
LRIT3

OFD1
CACNA1F
NYX
TREX1
ZNF423
RPGRIP1
C21orf2;
RB1;
CNGB3
RD3
AHI1
TRPM1
INVS, NPHP2;
RGS9
INPP5E, CORS1, JBTS1;
CABP4, CSNB2B;
PRPF3
TUBGCP6, MCCRP1;
DHX38
CEP78, C9orf81, CRDHL
PITPNM3
PRPF31
WHRN, DFNB31, USH2D;
IMPG2
VMD1/BEST1
BEST1, RP50, TU15B, VMD2;
GDF6
CLCC1
PLK4
TUB;
FAM161A
TTLL5
C8orf37/CFAP418
C1QTNF5, CTRP5;
CEP164, NPHP15;
NRL
CWC27
C12orf65, COXPD7, SPG55;
USH1G
BCAMD/ IMPG1

IMPG1
TULP1
ERCC6, ARMD5;
AGBL5
TMEM237
ARHGEF18
RP9
MAK
ESPN
AHR
POC5
PDZD7, PDZK7;
CNGB1
ZNF513
SLC24A1
ZNF408, EVR6, RP72;
ACBD5
RPGR
LCA5
PRDM13
PAX2, ONCR;
RAX2
RIMS1
GPR179
NEUROD1
RIMS2
COL9A1
COL11A1
HMX1
COL2A1, AOM, STL1;
TOPORs
KIAA1549
SAMD11
CSPP1
CRX
OTX2
RP1
VCAN
ATXN7
RP1L1

PCARE
SPATA7
ATF6
ALMS1
ARMS2, ARMD8, LOC387715;
PDE6H, ACHM6, RCD3, RCD3A;
PRCD
PDE6G
NDP
